# Multidimensional Optimal Power Flow with Voltage Profile Enhancement in Electrical Systems via Honey Badger Algorithm

**DOI:** 10.3390/biomimetics10120836

**Published:** 2025-12-14

**Authors:** Sultan Hassan Hakmi, Hashim Alnami, Badr M. Al Faiya, Ghareeb Moustafa

**Affiliations:** Department of Electrical and Electronics Engineering, Faculty of Engineering and Computer Science, Jazan University, Jizan 45142, Saudi Arabia; shhakmi@jazanu.edu.sa (S.H.H.); halnami@jazanu.edu.sa (H.A.); balfaiya@jazanu.edu.sa (B.M.A.F.)

**Keywords:** Optimal Power Flow, voltage profile enhancement, honey badger algorithm, electrical power networks

## Abstract

This study introduces an innovative Honey Badger Optimization (HBO) designed to address the Optimal Power Flow (OPF) challenge in electrical power systems. HBO is a unique population-based searching method inspired by the resourceful foraging behavior of honey badgers when hunting for food. In this algorithm, the dynamic search process of honey badgers, characterized by digging and honey-seeking tactics, is divided into two distinct stages, exploration and exploitation. The OPF problem is formulated with objectives including fuel cost minimization and voltage deviation reduction, alongside operational constraints such as generator limits, transformer settings, and line power flows. HBO is applied to the IEEE 30-bus test system, outperforming existing methods such as Particle Swarm Optimization (PSO) and Gray Wolf Optimization (GWO) in both fuel cost reduction and voltage profile enhancement. Results indicate significant improvements in system performance, achieving 38.5% and 22.78% better voltage deviations compared to GWO and PSO, respectively. This demonstrates HBO’s efficacy as a robust optimization tool for modern power systems. In addition to the single-objective studies, a multi-objective OPF formulation was investigated to produce the complete Pareto front between fuel cost and voltage deviation objectives. The proposed HBO successfully generated a well-distributed set of trade-off solutions, revealing a clear conflict between economic efficiency and voltage quality. The Pareto analysis demonstrated HBO’s strong capability to balance these competing objectives, identify knee-point operating conditions, and provide flexible decision-making options for system operators.

## 1. Introduction

The increasing complexity and interconnectivity of electrical power systems necessitate innovative approaches to enhance efficiency, reliability, and sustainability. Among these approaches, Optimal Power Flow (OPF) emerges as a cornerstone for addressing the multi-faceted challenges in energy systems [1,2,3]. It entails figuring out a power system’s ideal operating conditions while respecting system limitations and accomplishing predetermined goals, such as minimizing generation costs, reducing power losses, or integrating renewable energy sources [4]. It serves as a critical tool for addressing these issues by enabling system operators to make informed decisions that optimize resource allocation, enhance system stability, and meet environmental targets. A diverse range of classical optimization methods has been employed to address OPF problems, focusing primarily on single-objective formulations. These methods include the gradient projection method (GPM), linear programming, Newton-based approaches, nonlinear programming, quadratic programming, and interior-point methods [5,6]. While these techniques have proven effective in certain scenarios, their reliance on problem simplifications often leads to convergence at local optima rather than the global solution. In contrast, the economic dispatch model is a simplified model of the OPF problem that is concerned with only the real power outputs of the generators as control variables [7,8]. The dispatch problem is also extended to integrate cogeneration units to produce electrical and thermal energies [9,10].

In recent years, modern optimization algorithms have gained traction for solving OPF problems, offering greater flexibility and robustness. Techniques such as Genetic Algorithm (GA) [11], Particle Swarm Optimizer (PSO) [12], Differential Evolution (DE) [13], Crow Search Algorithm (CSA) [14], pigeon-inspired optimization [15], teaching-learning-studying optimizer [16], social network search algorithm [17], Gorilla troops algorithm [18] and moth swarm algorithm [19]. In [20], an evolutionary-inspired Pelican optimization has been designed for solving the OPF problem. The performance of POA is assessed on the IEEE 0-bus test system, addressing multiple objectives such as minimizing fuel costs, enhancing voltage profiles, and improving voltage stability. In [21], a Harris Hawks Optimization (HHO), inspired by the predatory strategies and cooperative behavior of Harris hawks, has emerged to solve the OPF. This study focused on optimizing key objective functions in OPF, such as minimizing fuel costs, reducing emissions, lowering active power losses, and minimizing voltage deviations across system buses with the application on the IEEE 57-bus power system. In [22], the Zebra Optimization Algorithm (ZOA) has been introduced to solve the OPF problem effectively on the standard IEEE 30-bus test system.

Also, several hybridized optimization techniques have emerged as a powerful approach to addressing the limitations of classical optimization methods, particularly in solving complex, real-world problems. Hybrid optimization techniques aim to overcome the challenges of conventional methods by combining the strengths of different algorithms, creating a synergy that enhances their overall performance and adaptability [23]. The hybridization process typically involves integrating global search algorithms, such as evolutionary strategies or swarm intelligence, with local search techniques or mathematical programming methods [24]. This combination ensures a balance between exploration and exploitation, enabling the algorithm to search broadly for potential solutions while refining them to achieve optimality. In [25], a Hybrid Firefly PSO (HFPSO) algorithm has been utilized to address nonlinear OPF problems. The HFPSO algorithm combines Firefly Optimization (FFO) and PSO to enhance both exploration and exploitation capabilities while accelerating convergence with application to five OPF objectives on the IEEE 30-bus test system. In [26], a novel hybridized algorithm combining HHO and DE (HHODE) was designed to enhance the capabilities of the standard HHO. By incorporating mutation operators from DE, HHODE improves the balance between exploration and exploitation, achieving better performance in optimization tasks. The algorithm’s effectiveness was demonstrated through a comparison with the original HHO using standard test benchmarks and the OPF with and without prohibited zones, using the IEEE 30-bus test system. Additionally, a hybrid technique integrating the whale optimizer and the slime mold algorithm has been introduced for the allocation of a hybrid power flow controller (HPFC) [27].

The Honey Badger Optimization (HBO) is rooted in the significant foraging techniques employed by honey badgers to obtain their food, such as digging for small prey or collaborating with honeyguide birds to locate beehives [28]. The first method, referred to as the “digging mode,” is executed solely by the honey badger, while the second, known as the “honey mode,” involves a cooperative effort between the badger and the birds. This cooperation is a demonstration of mutual benefit, leading to a balanced dynamic between exploration and exploitation throughout the search process. Since its inception, HBA has been applied to diverse real-world problems, showcasing its versatility. Early applications include feature selection in machine learning, where Hosny et al. integrated HBA with rough set approximations for enhanced classification accuracy [29]. In energy systems, Yakout et al. [30] and Han and Ghadimi [31] used HBA for parameter estimation in solid oxide fuel cells and proton-exchange membrane fuel cells, achieving lower error rates than traditional methods. Rawa et al. applied it to solar photovoltaic module modeling, outperforming other techniques in accuracy under varying conditions [32]. More recent works expand their scope, as summarized in Table 1.

This paper aims to design and apply HBO to solve the OPF problem, focusing on reducing fuel operating costs while enhancing the voltage profile of power systems. The approach is tested on the IEEE 30-bus standard test system. The main contributions of this work can be summarized as follows:

- A customized version of the HBO is developed specifically for OPF problems, with enhanced adaptation for OPF problems by penalty terms incorporation to guarantee the feasibility of the generated solutions regarding the operational limits.
- Extensive comparisons on the IEEE 30-bus system demonstrate that the proposed HBO achieves superior fuel cost minimization, voltage deviation reduction, and overall robustness compared to PSO and GWO.
- A detailed voltage profile assessment across all buses validates the operational advantages of HBO, showing significant improvements in voltage regulation.
- A multi-objective OPF framework utilizing a weighted-sum strategy is presented, allowing HBO to produce a well-distributed Pareto front that reflects the essential trade-off between fuel cost and voltage deviation.
- An in-depth Pareto-front analysis reveals knee-point operating regions, providing economic-voltage quality decision-making in practical power systems.

## 2. OPF Formulation

Optimal Power Flow (OPF) OPF involves determining the optimal operating conditions for a power system while adhering to system constraints and achieving specified objectives, such as minimizing the fuel production costs and improving the system voltage profile.

### 2.1. OPF Objectives

#### 2.1.1. Minimizing the Fuel Production Costs

One primary objective of OPF is to reduce the total fuel costs associated with power generation. The fuel cost for a generator is commonly represented using a quadratic polynomial model, expressed as:

(1) $$F_{1}=\sum_{i=1}^{N_{g}}a_{i}{\mathrm{Pg}}_{i}^{2}+b_{i}{\mathrm{Pg}}_{i}+c_{i}$$

where *F*_1_ denotes the total fuel cost in $/hr, *Pg_i_* denotes the MW active output power of generator *i*, and *a_i_*, *b_i_*, and *c_i_* are the cost coefficients for generator *i*. This formulation enables precise cost optimization across all generators in the system

#### 2.1.2. Enhancing the Voltage Profile

Voltage profile enhancement is a critical goal in OPF as it directly influences system stability and service quality. The aim is to minimize the deviation of bus load voltages from the desired reference value, typically set at 1 Per Unit (PU). The associated objective function is expressed as:

(2) $$F_{2}=\sum_{i=1}^{N_{\mathrm{Load}}}\left|V_{i}-V_{\mathrm{ref}}\right|$$

where *Vref* is the reference voltage of buses, which is taken as 1 PU, and *N_Load_* is the number of load buses. Here, *F*_2_ represents the total voltage deviation, *V_j_* is the voltage magnitude at load bus *j*, *V_ref_* is the reference voltage (1 PU), and *N_Load_* is the total number of load buses.

### 2.2. OPF Constraints

To guarantee the electrical power system operates effectively, it must satisfy a set of constraints. These include various operational limitations as inequality constraints that encompass:

- Limits on generated active power from the generators:

(3) $$P_{g\;i}^{\min}<P_{g\;i}<P_{g\;i}^{\max},\;\;i=1:N_{g}$$

- Limits on voltage settings control from the generators:

(4) $$V_{g}^{\min}\leq V_{g}\leq V_{g}^{\max},\;\;i=1:N_{g}$$

- Limits on transformer tap values:

(5) $$T_{k}^{\min}\leq T_{k}\leq T_{k}^{\max},\;\;k=1:N_{t}$$

- Limits on reactive power of existing *VAr* sources:

(6) $$0\leq QC_{k}\leq QC_{k}^{\max},\;\;k=1:N_{VAr}$$

- Limits on bus voltages:

(7) $$V_{i}^{\min}\leq V_{i}\leq V_{i}^{\max},\;\;i=1:N_{b}$$

- Limits on line power flow:

(8) $$\left|S_{L}^{flow}\right|\leq S_{L}^{\max},\;\;L=1:N_{L}$$

where *P_gi_*, *Q_gi_*, and *Vgi* denote the generated active power, reactive power, and voltage settings of each generation bus *i*.

The parameter *T_k_* represents the tap position of transformer *k*, where *N_t_* signifies the total count of transformers equipped with on-load tap-changing capabilities within the system. Additionally, *QC_k_* corresponds to the reactive power output provided by the *VAr* compensation device connected at bus *k*, and *N_Var_* indicates the total number of *VAr* sources present in the system. The variable *S_L_* denotes the apparent power flowing through transmission line *L*, while *N_L_* encompasses the complete set of transmission lines in the network.

## 3. HBO Algorithm for OPF

To tackle the OPF problem, this paper introduces a new design for HBO. In the algorithm, the dynamic searching process is divided into two phases: the exploration phase, focused on finding diverse solutions, and the exploitation phase, aimed at refining the best solutions [28]. The honey badger uses “digging” and “honey” modes to search for prey and honey, demonstrating the mutual benefits of collaboration between the badger and honeyguide birds.

The algorithm initializes honey badger populations based on their positions in the search space, calculates search intensity based on prey location, and controls the transition from exploration to exploitation as follows [44]:

(9) $$Y_{j}=Xd_{1}\times \left(upb_{j}-lob_{j}\right)+\;lb_{j}$$

where *Xd*_1_ is an arbitrary value that ranges from 0 to 1, *Y_j_* indicates the location of each badger (i) in the population number (*Ns*), and *lob_j_* and *upb_j_* represent the bottom and upward bounds of every location in the query space.

The intensity of the search, related to the prey’s location, is calculated based on the distance between the honey badger and the prey, as well as the strength of the signal, as follows [28]:

(10) Ij=Xd2×SST4π×YPR−Yj2

(11) $$SST={\left(Y_{j}-Y_{j+1}\right)}^{2}\;$$

whereas *SST* denotes source strength; *Xd*_2_ refers to an arbitrary value that ranges from 0 to 1; *Y_PR_* and *Y_j_* indicate the locations of, respectively, the prey and the *j*th badger. A density factor decreases over time, and a flag is introduced to modify search direction for more thorough exploration [32]:

(12) $$\phi =C1\times e\left(\frac{-TR}{TR_{max}}\right)\;$$

where *ϕ* symbolizes the density factor; *C*1 is a constant; *TR* and *TR_max_* are, respectively, the current and highest iteration number. Additionally, the flag (*Fg*) can be represented as follows:

(13) $$Fg=\left\{\begin{array}{c} 1\;\;\;\;if\;Xd_{3}\leq 0.5\\ -1\;\;\;\;\;if\;Xd_{3}>0.5 \end{array}\;\right.$$

where *Xd*_3_ refers to an arbitrary value that ranges from 0 to 1.

The positions of the honey badgers are updated in two phases: the “digging” phase and the “honey” phase. In the digging phase, the honey badger’s movement follows a cardioid-like pattern, influenced by various parameters, including the prey’s position and random factors [45]:

(14) $$Y_{N,j}=Y_{PR}+C2\times Fg\times I_{j}\times Y_{prey}+AB_{j}\;$$

(14a) $$AB_{j}=Xd_{4}\times \phi \times Fg\times \left(Y_{PR}-Y_{j}\right)\times Z$$

(14b) $$Z=\left|\cos\left(2\pi Xd_{5}\right)\times \left[1-\cos\left(2\pi Xd_{6}\right)\right]\right|\;$$

whereas *Xd*_4_, *Xd*_5_, and *Xd*_6_ denote arbitrary values that range from 0 to 1, while *C*2 is a constant representing the ability of honey badgers to achieve the food.

**Figure 1:**
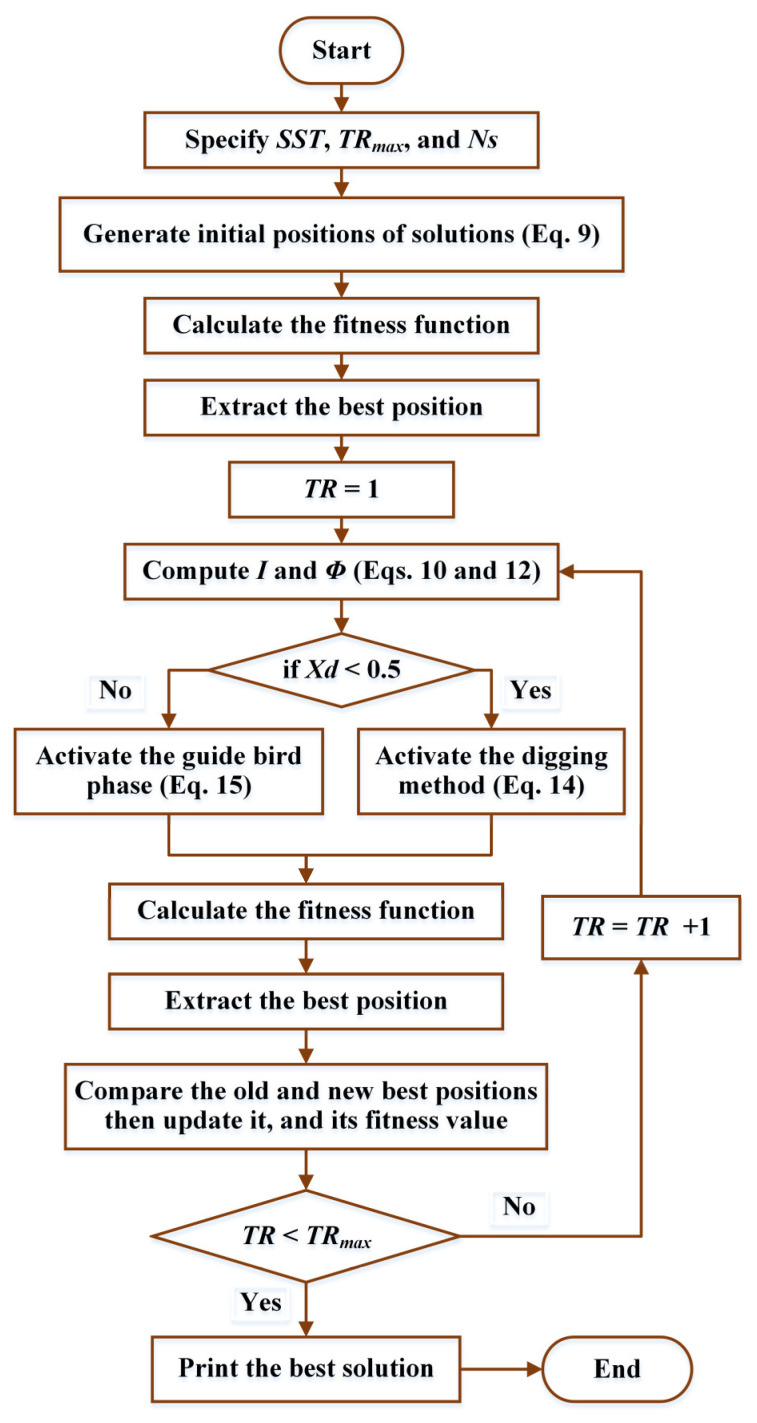
HBO Flowchart.

In the honey phase, the badger cooperates with the honeyguide bird to reach the beehive, adjusting its movement accordingly. These phases help the algorithm maintain an effective balance between exploring new areas and refining existing solutions [46]:

(15) $$Y_{N}=Y_{PR}+Xd_{7}\times \phi \times Fg\times \left(Y_{PR}-Y_{j}\right)$$

where the value of the random number *Xd*_7_ ranges from 0 to 1, Figure 1 displays the HBOs flowchart schematic.

In OPF problems, the HBO must search within a feasible region defined by equality constraints (power flow equations) and inequality constraints (operational limits). Since HBO cannot enforce these constraints analytically, a constraint-handling mechanism is incorporated into the objective evaluation. In this study, the constraints are handled through a Penalty Function Method, which transforms the constrained optimization problem into an unconstrained one by adding penalty terms to the objective function whenever violations occur.

For each candidate solution generated by HBO, the augmented objective function is formulated as:

(16) $$F=F_{\mathrm{objective}}+\underset{k}{\sum }\lambda_{k}\cdot {\mathrm{Violation}}_{k}$$

where $F_{\mathrm{objective}}\;$ is the primary objective, which can be the fuel cost in Case 1 or the voltage deviation in Case 2; ${\mathrm{Violation}}_{k}$. represents the amount by which a constraint is violated; $\lambda_{k}\;$ it is a penalty coefficient that determines the severity of penalization. This formulation ensures that the feasible solutions with no violations will have a low objective value, while the infeasible solutions that have a penalty term become large, and so they are automatically rejected. Thus, HBO is guided toward feasible regions of the search space.

The OPF model includes several inequality constraints. Each constraint is converted into a violation term. The major constraints penalized are (i) the bus voltage magnitude violations, (ii) the generator active power limits, (iii) the generator reactive power limits, and (iv) the transmission line flow limits. Additionally, the penalty coefficients $\lambda_{k}\;$. are chosen large enough to force HBO to favor feasible solutions. These penalties are added only if a violation occurs; otherwise, they contribute zero. This strategy ensures that feasibility is strictly enforced without interrupting the natural exploration–exploitation mechanism of HBO.

## 4. Simulation Results

**Figure 2:**
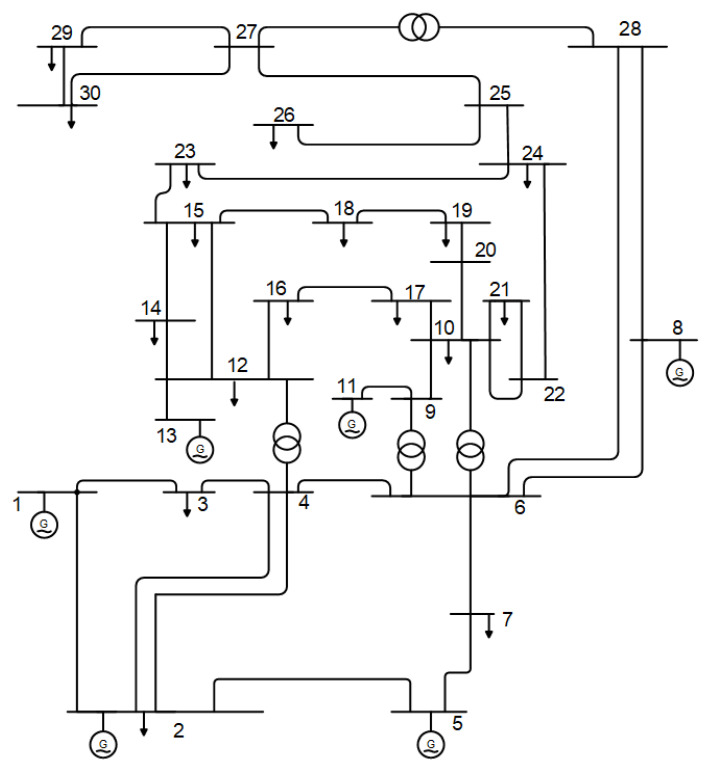
IEEE 30-bus network.

The performance and effectiveness of the proposed HBO method to solve the OPF problem are evaluated by considering the standardized IEEE 30-bus electrical system. The developed HBO is used for 20 simulation runs in comparison to PSO and GWO. Table 2 tabulates the parameter settings used by the three compared algorithms. From Table 2, the three compared algorithms have a similar number of function evaluations of 15,000 times, ensuring fair comparison. Figure 2 depicts the standardized system under investigation, which consists of 41 transmission lines, 30 buses, four tap changers, six generators, and nine reactive power devices [47,48]. The highest and lowest values of generator voltages are 1.1 and 0.95 PU, respectively.

The study evaluates two distinct scenarios, described as follows:

- Case 1 (*F*_1_ minimization): Focuses on minimizing the fuel production costs in Equation (1).
- Case 2 (*F*_2_ minimization): Aims to minimize the system voltages’ deviation in Equation (2).

### 4.1. Case 1: Fuel Costs Minimization

**Figure 3:**
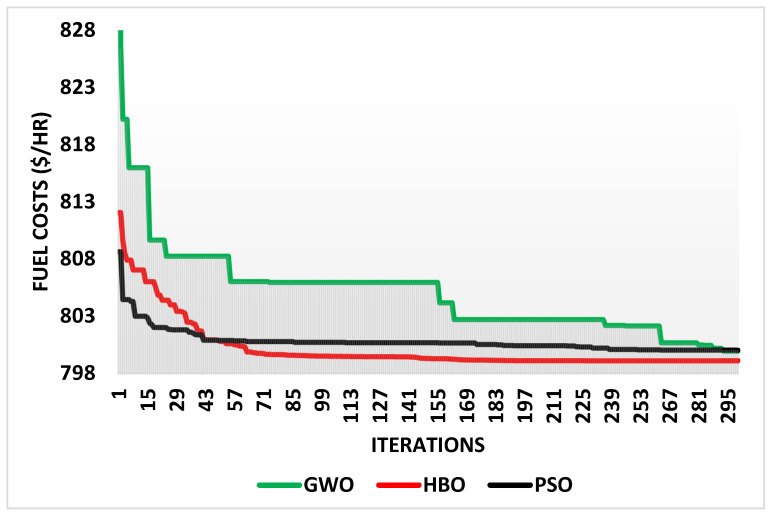
Convergences of GWO, HBO, and PSO for Case 1.

In contrast to the widely used PSO and GWO algorithms, the suggested HBO has been run for Case 1. Table 3 displays the control variables’ ideal results and the associated losses, and Figure 3 shows the convergences of GWO, HBO, and PSO for Case 1.

According to Table 3’s statistics, HBO outperforms both GWO and PSO in terms of fuel costs. While GWO attains fuel expenses of 799.96 $/hr and PSO achieves fuel costs of 800.023 $/hr, the proposed HBO lowers the fuel costs to 799.11$/hr. This indicates that, in contrast to GWO and PSO, HBO effectively reduces fuel expenses.

**Figure 4:**
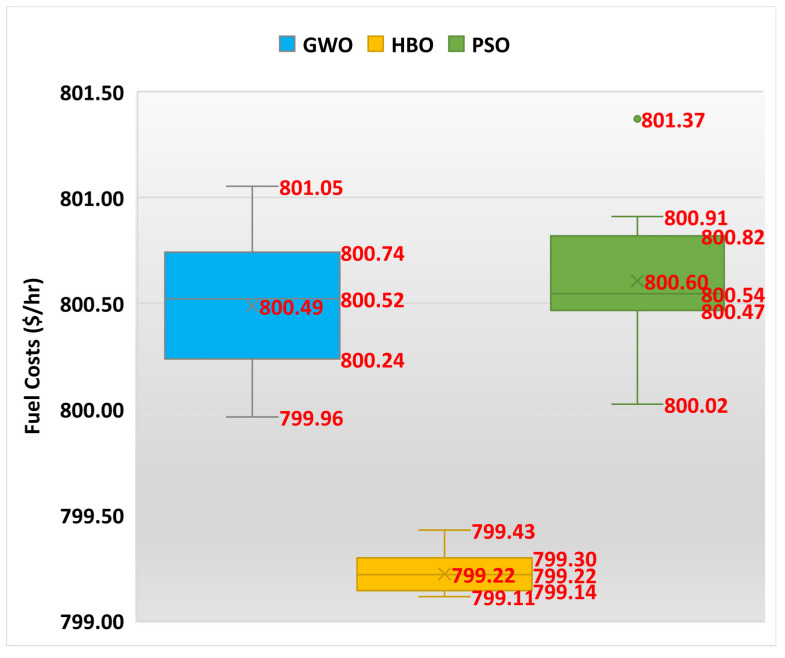
Boxplot of GWO, HBO, and PSO for Case 1.

Also, Figure 4 displays the boxplot of GWO, HBO, and PSO for Case 1. As shown, the designed HBO achieves the least maximum, mean, and minimum scores of power losses compared to GWO and PSO. According to the standard deviation, the proposed HBO attains a significant improvement percentage of 68.6% and 69.3% compared to GWO and PSO, respectively.

### 4.2. Case 2: System Voltages Deviation Minimization

**Figure 5:**
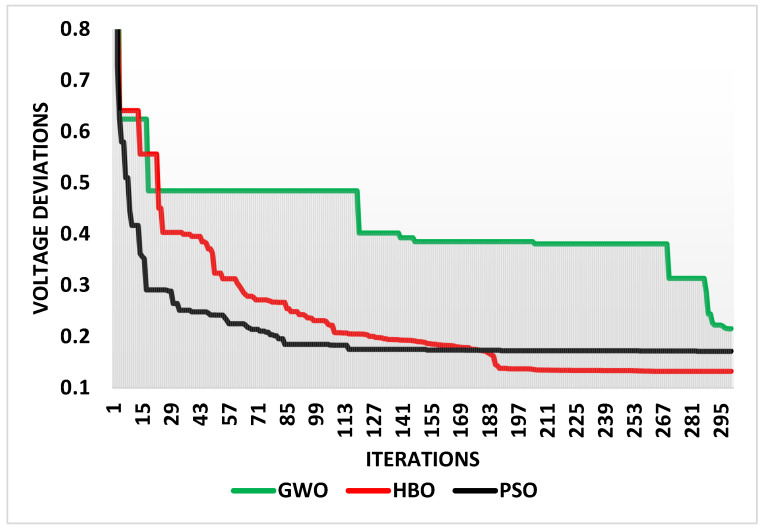
Convergences of GWO, HBO, and PSO for Case 2.

The proposed HBO algorithm has been run for Case 2 in contrast to the GWO and PSO. Their optimal outcomes of the control variables and their corresponding system voltage deviations are shown in Table 4, while Figure 5 depicts the convergences of GWO, HBO, and PSO for Case 2. As shown, HBO achieves the least voltage deviations, outperforming both GWO and PSO. HBO reduces the system voltage deviations to 0.132 while GWO and PSO achieve counterparts of 0.215 and 0.171. Therefore, HBO reduces the voltage deviations with better improvement, with 38.5% compared to the GWO and 22.78% compared to the PSO.

**Figure 6:**
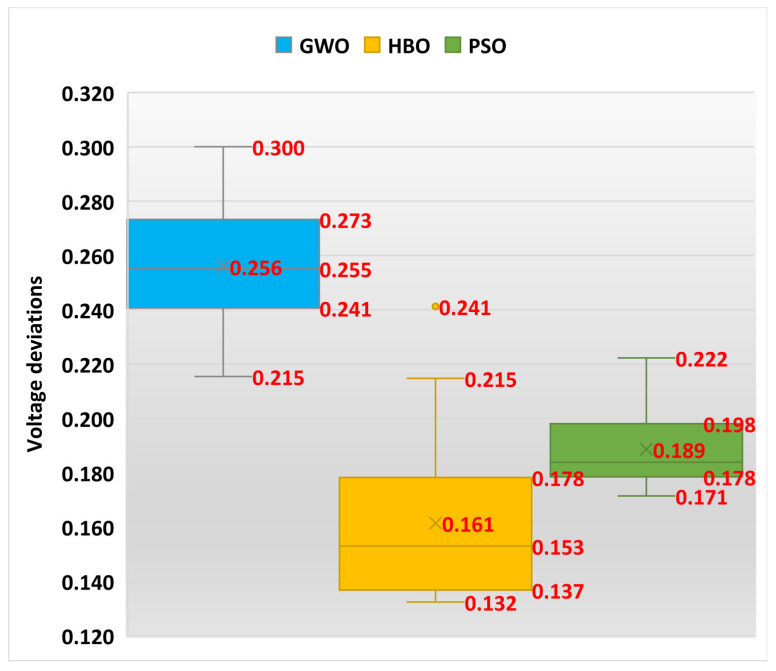
Boxplot of GWO, HBO, and PSO for Case 2.

Also, Figure 6 displays the boxplot of GWO, HBO, and PSO for Case 2. As shown, the designed HBO achieves the least maximum, mean, and minimum scores of power losses compared to GWO and PSO. According to the mean system voltage deviations, the proposed HBO attains a significant improvement percentage of 36.92% and 14.42% compared to GWO and PSO, respectively.

### 4.3. Analysis of Voltage Magnitudes at Each Bus for the IEEE 30-Bus System

**Figure 7:**
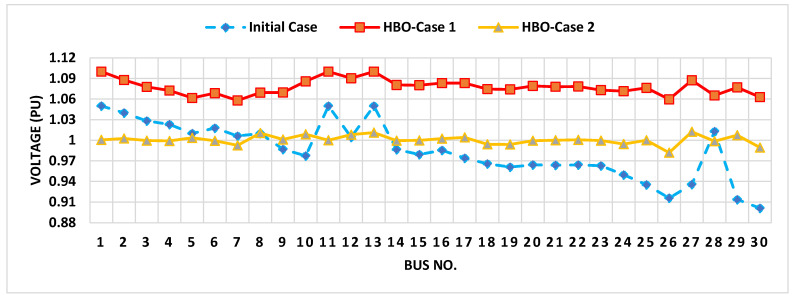
Voltage Profiles of the proposed HBO for Cases 1 and 2 compared to the initial one.

The proposed HBO algorithm has been run for Cases 1 and 2 with high superiority compared to GWO and PSO, as previously stated. To analyze the voltage impacts at each bus. Figure 7 displays the analysis of voltage magnitudes at each bus for the IEEE 30-bus system at Cases 1 and 2, in contrast to the initial case.

As shown, the voltage magnitudes in the initial case are generally lower than those in the HBO cases, with several buses below 1.0 PU, indicating worse voltage regulation. The HBO-Case 1 achieves significant improvements, with most bus voltages approaching or exceeding 1.0 PU, aligning with the preferred value. The HBO-Case 2 also improves voltage levels but maintains a tighter range around 1.0 PU, especially for buses that were closer to 1.0 in the initial case.

As shown, the average voltage in the initial case is 0.985 PU; in HBO-Case 1, it is 1.078 PU, while in HBO-Case 2, it is 1.001 PU. In addition, there are 14 buses which are operating below 1.0 PU in the initial case, while there are only two buses similar to those that are slightly below 1.0 in the HBO-Case 2. Moreover, the maximum operating voltages are 1.05 PU, 1.1 PU, and 1.0105 PU, respectively, in the initial case, HBO-Case 1, and HBO-Case 2. Overall, the HBO-Case 2 is more conservative, prioritizing values closer to 1.0 PU, aligning better with the desired range for system stability.

To address the multidimensional nature of the OPF problem, this study considers the two conflicting objectives simultaneously: the minimization of fuel generation costs (Equation (1)) and the reduction in system voltage deviations (Equation (2)). Because these objectives naturally compete, where lower operational cost tends to push the system closer to its limits, while a high-quality voltage profile often requires more expensive dispatch, the optimization must determine a balanced compromise rather than a single optimal point.

To capture this balance, the multi-objective OPF is transformed into a scalar optimization problem using the weighted-sum method, a widely accepted technique that allows the optimizer to explore different trade-off regions by assigning a tunable weight to each objective. The aggregated objective function is formulated as:

(17) $$F_{total}=W\times F_{1}+\left(1-W\right)\times F_{2}$$

Here, W  represents the weighting factor applied to the fuel cost objective $F_{1}$, while $\left(1-W\right)\;$ scales the voltage deviation objective $F_{2}$. By adjusting W, the decision maker can explicitly control the priority given to each objective. When W is set to one, the optimization focuses predominantly on fuel cost minimization, aligning with economic efficiency goals. When W is set to zero, greater emphasis is placed on voltage profile enhancement, supporting system stability, and power-quality considerations.

**Figure 8:**
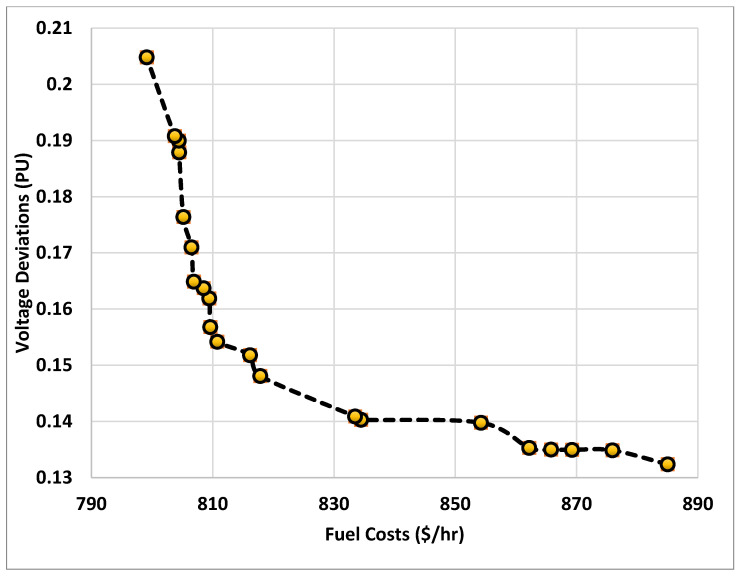
Pareto front of the suggested HBO for multi-objective OPF with the Weighted Sum approach.

In this study, the proposed HBOT algorithm is executed for 21 weight factors, ranging from 0 to 1 in increments of 0.05. Each weight produces a distinct solution, and the collection of all non-dominated solutions yields the Pareto front, shown in Figure 8. This curve illustrates the intrinsic trade-off between fuel cost and voltage deviation, demonstrating how improving one objective inevitably degrades the other. As shown, the Pareto solutions show a smooth and monotonic trend. Solutions with excellent voltage profile (minimum VD ≈ 0.132 PU) correspond to high operating costs (≈880–890 $/hr) while solutions with low operating cost (≈799 $/hr, the global best from Case 1) exhibit higher voltage deviations (≈0.205 PU). From Figure 8, the knee region appears around fuel cost of 810–820 $/hr and voltage deviation of 0.15–0.16 PU, declaring solutions that achieve the best compromise. Additionally, HBO achieves dominant solutions comparable to single-objective runs where the Pareto point at 799.1132 $/hr, VD = 0.20482 matches the best cost-minimization result from Case 1. This confirms that HBO is consistent across single- and multi-objective formulations.

This analysis confirms that HBO is able to effectively navigate the conflicting objectives of OPF, producing practical, flexible, and diverse solutions suitable for real-world grid operation.

## 5. Conclusions

This research introduced HBO as an effective method for solving the OPF problem in electrical systems. The study focused on achieving two primary objectives: minimizing fuel production costs and enhancing the voltage profile while adhering to operational constraints. The proposed HBO was tested on the IEEE 30-bus standard system against PSO and GWO. The results unequivocally demonstrated HBO’s superior performance in both case studies. In the first case, focused on fuel cost minimization, HBO achieved the lowest fuel cost of 799.11 $/hr, outperforming GWO and PSO by significant margins. In the second case, aimed at minimizing voltage deviations, HBO achieved the smallest system voltage deviation of 0.132 PU, representing a 38.5% improvement over GWO and a 22.78% improvement over PSO. This highlights HBO’s capability to enhance voltage stability across the power system, ensuring more reliable and efficient operation. The analysis of voltage magnitudes across individual buses further validated HBO’s effectiveness. Compared to the initial case, HBO significantly improved the voltage profile, bringing most bus voltages closer to the preferred value of 1.0 PU while reducing the number of buses operating below 1.0 PU. This is particularly evident in Case 2, where the algorithm prioritized stability by maintaining voltage magnitudes within a tighter and more desirable range. Beyond the single-objective evaluations, the proposed HBO also proved highly effective in handling the multi-objective OPF formulation through a weighted-sum strategy. The resulting Pareto front revealed a distinct and well-structured trade-off between fuel cost and voltage deviation. HBO produced smoothly distributed Pareto-optimal solutions, including a clear knee region that offers an attractive compromise between economic efficiency and voltage quality. This capability enables system operators to select operating points based on real-time priorities and operational constraints. The strong multi-objective performance further validates HBO as a versatile and powerful optimization tool for modern power system planning and operation.

Although the proposed HBO framework demonstrated strong performance, the study is limited to the IEEE 30-bus system. The weighted-sum approach, while effective, may miss certain non-convex regions of the Pareto front. Additionally, the HBO parameters were effectively tuned; this may influence scalability for larger networks.

Future work could extend HBO to larger and more complex test systems, incorporate additional operational objectives such as emission reduction or renewable integration, and explore advanced multi-objective frameworks. Hybrid HBO versions combining local search operators and adaptive penalty mechanisms also represent promising directions to further enhance convergence and robustness.

## Figures and Tables

**Table 1:** Several recent applications of the HBO algorithm.

Application Domain	Specific Use	Reference	Year
Feature Selection	Enhanced feature selection by integrating rough set approximations	[29]	2022
Fuel Cell Modeling	Parameter estimations for solid oxide fuel cells	[30]	2022
Fuel Cell Modeling	Model identification for proton-exchange membrane fuel cells using hybrid CNN-ELM	[31]	2022
Solar Energy	Parameter estimation for solar photovoltaic modules	[32]	2022
Photovoltaic Systems	Parameter estimation for photovoltaic systems	[33]	2022
Wireless Sensor Networks	Clustering and routing protocol for energy efficiency	[34]	2023
Wireless Sensor Networks	Coverage improvement in sensor networks	[35]	2023
Load Frequency Control	frequency controller design for two-area systems with renewables	[36]	2023
Algorithm Enhancement	Lens opposition-based learning and local search for evolving systems	[37]	2024
Time Series Prediction	Predicting COVID-19 data using a multilayer perceptron	[38]	2024
Distribution Networks	Allocation of solar PV under uncertainty and load variation	[39]	2024
Wireless Sensor Networks	Multi-hop routing protocol for efficient and secure QoS	[40]	2024
Power Systems	Congestion management in power systems with renewable energy resources	[41]	2025
Fault Diagnosis	Fault diagnosis in processes using elite tangent search and differential mutation	[42]	2025
Distribution Grid Optimization	Optimal photovoltaic system placement in a probabilistic framework	[43]	2026

**Table 2:** Parameter settings of GWO, PSO and H.

Applied Algorithm	Parameter Settings
GWO	Number of gray wolves is 50; Number of iterations of 300.
PSO	Cognitive parameter is 2.05; Social parameter is 2.05; Number of particles is 50; Number of iterations of 300.
HBO	Constant (*C*1) in Equation (12) = 2; The ability of honey badgers to obtain the food (*C*2) in Equation (14) = 6; Search members of 50; Peak iterations of 300.

**Table 3:** Results of GWO, HBO, and PSO for Case 1.

Control Variable	GWO	HBO	PSO
Vg1	1.099939	1.100000	1.093945
Vg2	1.088631	1.087795	1.074532
Vg5	1.060868	1.061578	1.042043
Vg8	1.071014	1.069445	1.050394
Vg11	1.06387	1.100000	1.074047
Vg13	1.08699	1.100000	1.085673
T6–9	0.981534	1.057962	1.010207
T6–10	1.005407	0.900000	0.986821
T4–12	1.035632	0.992115	1.03323
T28–27	0.988049	0.966915	0.979331
Qc**_10_**	1.834642	5.000000	2.225086
Qc**_12_**	3.874838	5.000000	2.216019
Qc**_15_**	3.520814	4.977749	1.995828
Qc**_17_**	2.488012	5.000000	4.734907
Qc**_20_**	0.99104	5.000000	3.59593
Qc**_21_**	3.47999	5.000000	4.000956
Qc**_23_**	2.588832	0.000000	3.627154
Qc**_27_**	2.87374	5.000000	3.033964
Qc**_29_**	0.670334	2.542596	2.86218
Pg1	175.3163	177.0305	175.0281
Pg2	47.59272	48.68418	48.83014
Pg5	20.7254	21.32696	21.04257
Pg8	21.50606	21.08474	21.3284
Pg11	12.37276	11.90698	12.89795
Pg13	14.53182	12.000000	13.000000
Fuel Costs ($/hr)	799.9619	799.1132	800.0223

**Table 4:** Results of GWO, HBO, and PSO for Case 2.

Control Variable	GWO	HBO	PSO
Vg1	1.020727	1.000628	1.005525
Vg2	1.014141	1.002585	1.001557
Vg5	1.002397	1.003612	1.000364
Vg8	1.003282	1.010466	1.002322
Vg11	1.007813	1.000001	1.000712
Vg13	1.017874	1.011245	1.018207
T6–9	0.998105	1.008175	0.989971
T6–10	0.902382	0.900044	0.913218
T4–12	0.964564	1.000439	0.984205
T28–27	0.943213	0.974172	0.954124
Qc**_10_**	3.668575	2.513673	5.000000
Qc**_12_**	1.326187	5.000000	2.244941
Qc**_15_**	0.681326	5.000000	2.222555
Qc**_17_**	4.030658	4.964585	2.822482
Qc**_20_**	4.870814	4.991097	4.344489
Qc**_21_**	2.877625	5.000000	4.166829
Qc**_23_**	4.374506	5.000000	5.000000
Qc**_27_**	0.620946	4.986787	4.659375
Qc**_29_**	1.238647	5.000000	3.200829
Pg1	135.1729	53.73967	95.16623
Pg2	56.54347	80.000000	53.91632
Pg5	33.67601	50.000000	44.14318
Pg8	20.64266	35.000000	30.98693
Pg11	21.16756	28.43875	29.02789
Pg13	23.46645	40.000000	35.000000
Voltage deviations	0.215422	0.13238	0.171448

## Data Availability

Data is contained within the article. The original contributions presented in this study are included in the article. Further inquiries can be directed to the corresponding author(s).

## References

[1] Khamees A.K., Abdelaziz A.Y., Eskaros M.R., Alhelou H.H., Attia M.A. (2021). Stochastic Modeling for Wind Energy and Multi-Objective Optimal Power Flow by Novel Meta-Heuristic Method. IEEE Access.

[2] Sarhan S., Shaheen A.M., El-Sehiemy R.A., Gafar M. (2022). Enhanced Teaching Learning-Based Algorithm for Fuel Costs and Losses Minimization in AC-DC Systems. Mathematics.

[3] Alqahtani M.H., Almutairi S.Z., Shaheen A.M., Ginidi A.R. (2024). Enhanced Kepler Optimization Method for Nonlinear Multi-Dimensional Optimal Power Flow. Axioms.

[4] Premkumar M., Hashim T.J.T., Ravichandran S., Sin T.C., Chandran R., Alsoud A.R., Jangir P. (2024). Optimal operation and control of hybrid power systems with stochastic renewables and FACTS devices: An intelligent multi-objective optimization approach. Alex. Eng. J..

[5] Momoh J., Adapa R., El-Hawary M. (1999). A review of selected optimal power flow literature to 1993. I. Nonlinear and quadratic programming approaches. IEEE Trans. Power Syst..

[6] Fortenbacher P., Demiray T. (2019). Linear/quadratic programming-based optimal power flow using linear power flow and absolute loss approximations. Int. J. Electr. Power Energy Syst..

[7] Shaheen A.M., El-Rifaie A.M., Al Faiya B., Moustafa G., Alnami H. (2025). Economic and environmental optimization-dispatch in large-scale power systems using weighted mean of vectors algorithm. Sustain. Comput. Inform. Syst..

[8] Aljumah A.S., Alqahtani M.H., Shaheen A.M., Elsayed A.M. (2024). Enhanced Aquila Optimizer for Economic Environmental Dispatch with Cubic Fuel Cost and Emission Models. IEEE Access.

[9] Aljumah A.S., Alqahtani M.H., Ginidi A.R., Shaheen A.M. (2025). A novel Kangaroo Escape Algorithm for efficient combined heat and power economic dispatch: Feasibility analysis and validations. Energy Rep..

[10] Shaheen A.M., Ginidi A.R., Alassaf A., Alsaleh I. (2024). Developing artificial hummingbird algorithm with linear controlling strategy and diversified territorial foraging tactics for combined heat and power dispatch. Alex. Eng. J..

[11] Attia A.-F., Al-Turki Y.A., Abusorrah A.M. (2012). Optimal power flow using adapted genetic algorithm with adjusting population size. Electr. Power Compon. Syst..

[12] Syllignakis J.E., Kanellos F.D. (2021). A PSO Optimal Power Flow (OPF) Method for Autonomous Power Systems Interconnected with HVDC Technology. Electr. Power Compon. Syst..

[13] Guvenc U., Duman S., Kahraman H.T., Aras S., Katı M. (2021). Fitness–Distance Balance based adaptive guided differential evolution algorithm for security-constrained optimal power flow problem incorporating renewable energy sources. Appl. Soft Comput..

[14] Shaheen A.M., Elsayed A.M., El-Sehiemy R.A. (2021). Optimal Economic–Environmental Operation for AC-MTDC Grids by Improved Crow Search Algorithm. IEEE Syst. J..

[15] Chen G., Qian J., Zhang Z., Li S. (2020). Application of modified pigeon-inspired optimization algorithm and constraint-objective sorting rule on multi-objective optimal power flow problem. Appl. Soft Comput..

[16] Akbari E., Ghasemi M., Gil M., Rahimnejad A., Gadsden S.A. (2021). Optimal Power Flow via Teaching-Learning-Studying-Based Optimization Algorithm. Electr. Power Compon. Syst..

[17] El-Sehiemy R., Elsayed A., Shaheen A., Elattar E., Ginidi A. (2021). Scheduling of Generation Stations, OLTC Substation Transformers and VAR Sources for Sustainable Power System Operation Using SNS Optimizer. Sustainability.

[18] Ginidi A., Elattar E., Shaheen A., Elsayed A., El-Sehiemy R., Dorrah H. (2022). Optimal Power Flow Incorporating Thyristor-Controlled Series Capacitors Using the Gorilla Troops Algorithm. Int. Trans. Electr. Energy Syst..

[19] Mohamed A.-A.A., Mohamed Y.S., El-Gaafary A.A., Hemeida A.M. (2017). Optimal power flow using moth swarm algorithm. Electr. Power Syst. Res..

[20] Kumar P., Kalam A., Paul K. (2024). Optimal Power Flow Analysis Using Pelican Optimization Algorithm. Lecture Notes in Electrical Engineering.

[21] Al-Kaabi M., Dumbrava V., Eremia M. Application of Harris Hawks Optimization in Single Objective Optimal Power Flow. Proceedings of the 15th International Conference on Electronics, Computers and Artificial Intelligence, ECAI 2023.

[22] Choudhary J.K., Rohan R., Tanti D.K., Kumari N., Kumar P. Application of Zebra Optimisation Algorithm for Solving Optimal Power Flow Issues. Proceedings of the OCIT 2023—21st International Conference on Information Technology.

[23] Sabo A., Kanya K.I., Shu’aibu N., Onyema C., Aliyu A., Tanko H., Kwasau S. (2023). A Review of State-of-the-Art Techniques for Power Flow Analysis. J. Sci. Technol. Eng. Res..

[24] Gupta S., Kumar N., Srivastava L., Malik H., Marugán A.P., Márquez F.P.G. (2021). A hybrid jaya–powell’s pattern search algorithm for multi-objective optimal power flow incorporating distributed generation. Energies.

[25] Khan A., Hizam H., Wahab N.I.B.A., Othman M.L. (2020). Optimal power flow using hybrid firefly and particle swarm optimization algorithm. PLoS ONE.

[26] Birogul S. (2019). Hybrid harris hawk optimization based on differential evolution (HHODE) algorithm for optimal power flow problem. IEEE Access.

[27] Bhandakkar A.A.A., Mathew L. (2023). Merging slime mould with whale optimization algorithm for optimal allocation of hybrid power flow controller in power system. J. Exp. Theor. Artif. Intell..

[28] Hashim F.A., Houssein E.H., Hussain K., Mabrouk M.S., Al-Atabany W. (2022). Honey Badger Algorithm: New metaheuristic algorithm for solving optimization problems. Math. Comput. Simul..

[29] Hosny R.A., Elaziz M.A., Ibrahim R.A. (2022). Enhanced Feature Selection Based on Integration Containment Neighborhoods Rough Set Approximations and Binary Honey Badger Optimization. Comput. Intell. Neurosci..

[30] Yakout A.H., Kotb H., AboRas K.M., Hasanien H.M. (2022). Comparison among different recent metaheuristic algorithms for parameters estimation of solid oxide fuel cell: Steady-state and dynamic models. Alex. Eng. J..

[31] Han E., Ghadimi N. (2022). Model identification of proton-exchange membrane fuel cells based on a hybrid convolutional neural network and extreme learning machine optimized by improved honey badger algorithm. Sustain. Energy Technol. Assess..

[32] Rawa M., Abusorrah A., Al-Turki Y., Calasan M., Micev M., Ali Z.M., Mekhilef S., Bassi H., Sindi H., Aleem S.H.E.A. (2022). Estimation of Parameters of Different Equivalent Circuit Models of Solar Cells and Various Photovoltaic Modules Using Hybrid Variants of Honey Badger Algorithm and Artificial Gorilla Troops Optimizer. Mathematics.

[33] Diab A.A.Z., Tolba M.A., El-Rifaie A.M., Denis K.A. (2022). Photovoltaic parameter estimation using honey badger algorithm and African vulture optimization algorithm. Energy Rep..

[34] Arutchelvan K., Priya R.S., Bhuvaneswari C. (2023). Honey Badger Algorithm Based Clustering with Routing Protocol for Wireless Sensor Networks. Intell. Autom. Soft Comput..

[35] Luo Y., Hu Y. (2023). The Coverage Improvement of the Wireless Sensor Network Based on the Parameters Optimized Honey Badger Algorithm. IEEE Access.

[36] Ozumcan S., Ozturk A., Varan M., Andic C. (2023). A novel honey badger algorithm based load frequency controller design of a two-area system with renewable energy sources. Energy Rep..

[37] Majumdar P., Mitra S., Bhattacharya D. (2024). Honey Badger algorithm using lens opposition based learning and local search algorithm. Evol. Syst..

[38] Qasem S.N. (2024). A novel honey badger algorithm with multilayer perceptron for predicting COVID-19 time series data. J. Supercomput..

[39] Hareche M.L., Ladjici A.A. (2024). Optimal allocation of solar photovoltaic distributed generation for performance enhancement of electrical distribution networks considering optimal volt-var regulation under uncertainty and high load variation. Optim. Control Appl. Methods.

[40] Altuwairiqi M. (2024). An optimized multi-hop routing protocol for wireless sensor network using improved honey badger optimization algorithm for efficient and secure QoS. Comput. Commun..

[41] Duong V.T., Duong T.L. (2025). An Improved Honey Badger Algorithm for Solving Congestion Management of Power System Considering Effective of Renewable Energy Resources. Int. J. Intell. Eng. Syst..

[42] Ting H., Yong C., Peng C. (2025). Improved Honey Badger Algorithm Based on Elite Tangent Search and Differential Mutation with Applications in Fault Diagnosis. Processes.

[43] Barutcu I.C., Sharma G., Sant A.V., Kumar R. (2026). Optimal photovoltaic system placement on the distribution grid by applying modified honey badger algorithm in the probabilistic framework. Energy Convers. Manag..

[44] El-Sehiemy R., Shaheen A., Ginidi A., Elhosseini M. (2022). A Honey Badger Optimization for Minimizing the Pollutant Environmental Emissions-Based Economic Dispatch Model Integrating Combined Heat and Power Units. Energies.

[45] El-Ela A.A.A., El-Sehiemy R.A., Shaheen A.M., Shalaby A.S., Mouwafi M.T. (2024). Robust generation expansion planning in power grids under renewable energy penetration via honey badger algorithm. Neural Comput. Appl..

[46] El Ela A.A.A., El-Sehiemy R.A., Shaheen A.M., Shalaby A.S., Mouafi M.T. (2023). Reliability constrained dynamic generation expansion planning using honey badger algorithm. Sci. Rep..

[47] Sarhan S., El-Sehiemy R.A., Shaheen A.M., Gafar M. (2023). TLBO merged with studying effect for Economic Environmental Energy Management in High Voltage AC Networks Hybridized with Multi-Terminal DC Lines. Appl. Soft Comput..

[48] Shaheen A.M., El-Sehiemy R.A., Farrag S.M. (2016). Optimal reactive power dispatch using backtracking search algorithm. Aust. J. Electr. Electron. Eng..

